# Biomass-Derived Porous Carbon-Based Nanostructures for Microwave Absorption

**DOI:** 10.1007/s40820-019-0255-3

**Published:** 2019-03-15

**Authors:** Huanqin Zhao, Yan Cheng, Wei Liu, Lieji Yang, Baoshan Zhang, Luyuan Paul Wang, Guangbin Ji, Zhichuan J. Xu

**Affiliations:** 1 College of Materials Science and Technology, Nanjing University of Aeronautics and Astronautics, Nanjing, 210016 People’s Republic of China; 2 School of Electronic Science and Engineering, Nanjing University, Nanjing, 210093 People’s Republic of China; 3 School of Materials Sciences and Engineering, Nanyang Technological University, 50 Nanyang Avenue, Singapore, 639798 Singapore; 4 Singapore-HUJ Alliance for Research and Enterprise, NEW-CREATE Phase II, Campus for Research Excellence and Technological Enterprise (CREATE), Singapore, 138602 Singapore

**Keywords:** Biomass resource, Porous carbon, Microwave absorption

## Abstract

Currently, electromagnetic (EM) pollution poses severe complication toward the operation of electronic devices and biological systems. To this end, it is pertinent to develop novel microwave absorbers through compositional and structural design. Porous carbon (PC) materials demonstrate great potential in EM wave absorption due to their ultralow density, large surface area, and excellent dielectric loss ability. However, the large-scale production of PC materials through low-cost and simple synthetic route is a challenge. Deriving PC materials through biomass sources is a sustainable, ubiquitous, and low-cost method, which comes with many desired features, such as hierarchical texture, periodic pattern, and some unique nanoarchitecture. Using the bio-inspired microstructure to manufacture PC materials in mild condition is desirable. In this review, we summarize the EM wave absorption application of biomass-derived PC materials from optimizing structure and designing composition. The corresponding synthetic mechanisms and development prospects are discussed as well. The perspective in this field is given at the end of the article.

## Highlights

- The synthetic methods and corresponding mechanisms of porous carbon (PC)-based nanostructures from biomass resource are reviewed.
- The application of biomass-derived PC in microwave absorption is discussed in terms of structure and composition optimization.

## Graphical abstract

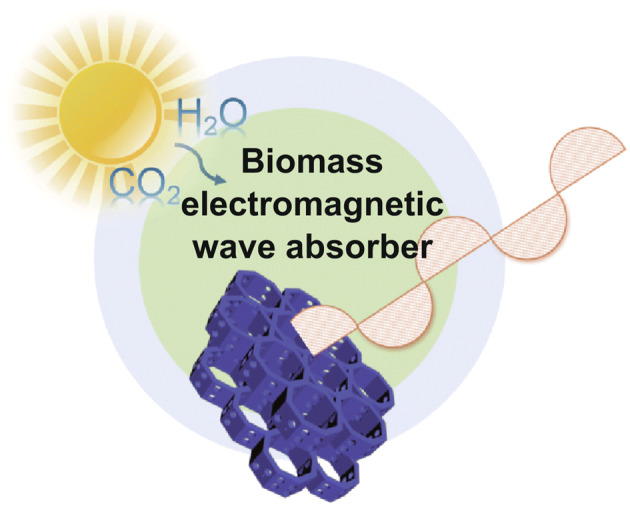

## Introduction

The rapid development of electronic technologies brings about great convenience to humans’ life. However, excessive usage of electronics also results in serious electromagnetic (EM) radiation and interference, which is detrimental to human health [1–5]. Consequently, the research on use of functional EM absorbers to eliminate the unwanted EM energies has attracted significant attention. Among the reported materials, carbon-based nanomaterials are promising because of their adjustable dielectric properties, low density, and good environmental stability [6–9].

Over the past decades, various carbon-based nanomaterials have emerged as ideal candidates due to their lightweight as well as outstanding EM wave absorption capabilities. In particular, the successful employment of graphene and carbon nanotubes (CNT) in EM wave absorption field brought great development, due to their high electrical conductivity, low percolation threshold, and special nanostructure [10–13]. In recent years, there have been booming studies based on CNT and graphene [14–18]. Unfortunately, synthesis of such materials requires expensive raw materials (e.g., fossil) and is subjected to energy-intensive processes (chemical vapor deposition (CVD), hummers methods arc discharge, etc.) [19, 20]. These unavoidable shortcomings hinder their practical application. Thus, exploring sustainable and economic raw materials to produce versatile carbon materials, accompanied with a facile synthesis technology, is highly desired.

**Fig. 1:**
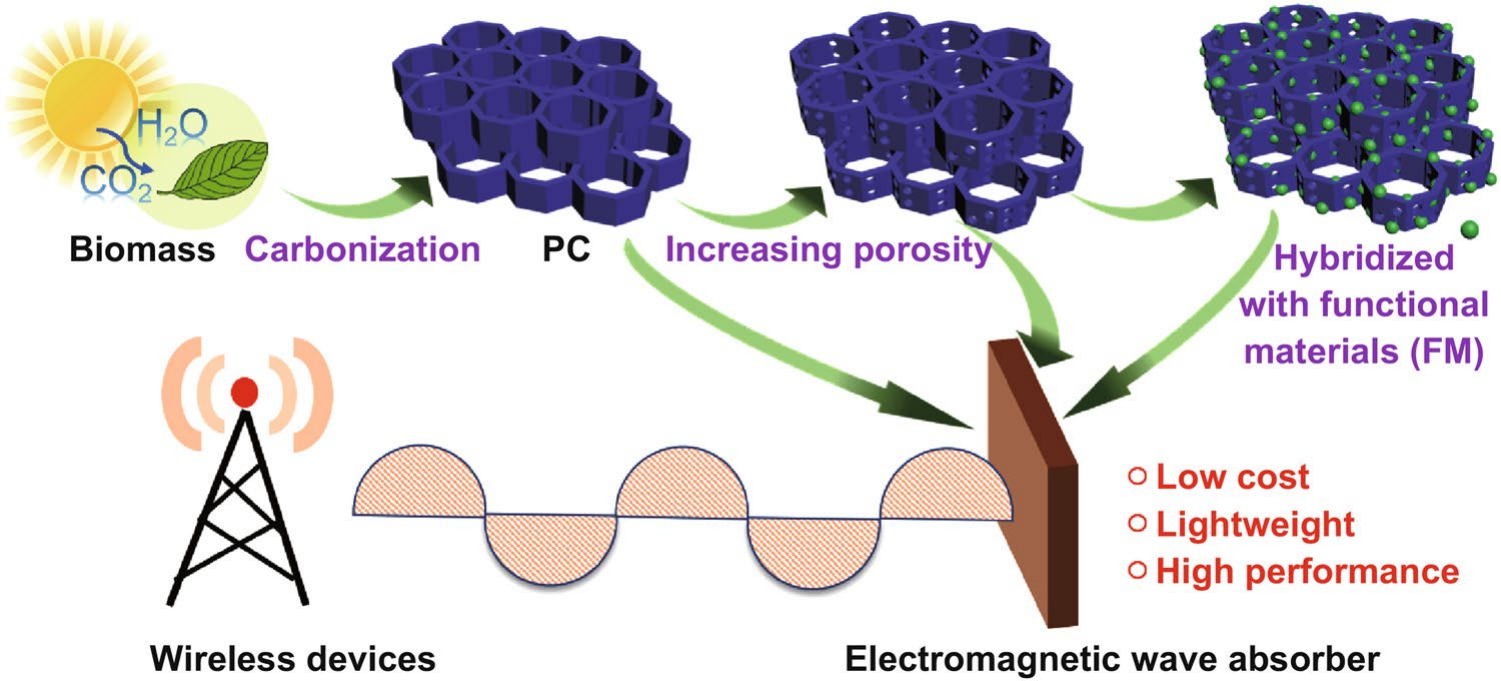
Schematic representation of biomass derive PC in EM absorption application

Biomass is renewable, eco-friendly, and abundant resource present around the world [21–23]. After various human agricultural activities, abundant biomass residues can be easily found. However, large amounts of agricultural resides and forest byproducts are directly discarded or incinerated, leading to severe environmental damage [24, 25]. The use of low-cost biomass residues as raw materials to fabricate carbon-based absorber is an environmentally friendly and promising route. Recent reports have revealed that the porous structure is beneficial for enhancing EM wave absorption [26–30]. The presence of pores not only decreases the bulk density, but also improves impedance matching of absorber [31, 32]. Interestingly, biomass in nature has many desired characteristics, for example, elaborate periodic porous microstructure and microtubular channels [33, 34]. As mentioned above, the favorable porous structures have close relationship with properties of EM wave absorption and attenuation. By utilizing biomass as raw materials, the porous carbon (PC) could be prepared through a simple thermal treatment process [35]. As a result, biomass-derived PC has been considered as a novel and economically viable mean for dealing with EM pollution. Until now, tremendous trials have been dedicated to the exploitation of biomass-derived PC in EM absorption, including optimizing pore size, enlarging surface area, and constructing multicomponent. In this progress report, we comprehensively summarize recent progress in rational design and fabrication of novel PC-based absorber from biomass source and systematically discuss the key factors influencing the EM wave absorption performance (pore architecture and compositions), as shown in Fig. 1. We expect this timely review to provide a constructive and general guideline for the design of novel absorber from biomass resource.

## The Role of Porous Structure in Microwave Absorption

Numerous previous literatures have confirmed that porous structure plays a positive role in attenuating EM energies [36–39]. It is well accepted that porous materials could be regarded as a composite, comprising of solid media (host) and air (inclusion) [29]. Many models have been established to describe the dielectric properties of composite in numerical fits, analytic derivations, and stochastic methods. There are typically three models based on host-inclusion medium, containing Bruggeman 1/3 power law, Landauer–Bruggeman effective medium approximation (EMA), and Maxwell-Garnet (MG) [40, 41]. With reference to Wang’s work [40], they prepared porous titania and calcium magnesium with different porosities and investigated the influence of porosity on complex permittivity (*ε*_*r*_). It is concluded that *ε*_*r*_ values decrease with porosity increasing in both porous titania and calcium magnesium. The change tendency is almost complied with the MG, EMA, and Bruggeman 1/3 power law. Among these models, the MG theory has been widely applied in diverse porous materials, and it can be expressed as Eq. 1 [42, 43]:

1 $$\varepsilon_{\text{eff}}^{\text{MG}} = \left[ {\frac{{(\varepsilon_{2} + 2\varepsilon_{1} ) + 2f_{r} (\varepsilon_{2} - \varepsilon_{1} )}}{{(\varepsilon_{2} + 2\varepsilon_{1} ) - f_{r} (\varepsilon_{2} - \varepsilon_{1} )}}} \right]\varepsilon_{1}$$

where *ε*_1_ and *ε*_2_ are the permittivity of solid and free air and *f*_r_ relates to the volume percentage of air in the effective medium. From this formula, we can conclude that the presence of a porous architecture will reduce complex permittivity. In general, the impedance matching and EM attenuation capacity are fundamental design principles for an absorber. The poor attenuation capacity would result in weak microwave absorption intensity. Similarly, the inferior impedance matching would give rise to the reflection of EM wave on the absorber surface. Hence, the optimal impedance matching and strong EM attenuation competence are desired for an excellent absorber. The ideal impedance matching requires that the characteristic impedance of material (*Z*_im_) is close to that of free air (*Z* = 1) [44, 45]. The characteristic impedance is expressed by the relative complex permittivity (*ε*_*r*_) and complex permeability (*μ*_*r*_) (Eq. 2) [46]:

2 $$Z_{\text{im}} = \sqrt {\frac{{\mu_{0} }}{{\varepsilon_{0} }}} \sqrt {\frac{{\mu_{r} }}{{\varepsilon_{r} }}}$$

where *ε*_0_ and *μ*_0_ are the relative complex permittivity and permeability of vacuum, respectively. As we know, *ε*_*r*_ is usually larger than *μ*_*r*_ for any absorber. So decline of *ε*_*r*_ would result in the *Z*_im_ value of near 1 [47]. Combined with the above analysis, constructing porous structure is an efficient strategy to improve impedance matching of material, because of decreases in effective permittivity. Additionally, the porous structure would generate abundant solid–air boundaries in the interior of media. When the extra EM wave radiates on these boundaries, plenty of charges would accumulate at the carbon–air interfaces, inducing the strong space charge polarization. This can boost the EM wave attenuation capacity of material [48].

## Pure PC Absorber from Biomass

### Direct Pyrolysis Method

**Fig. 2:**
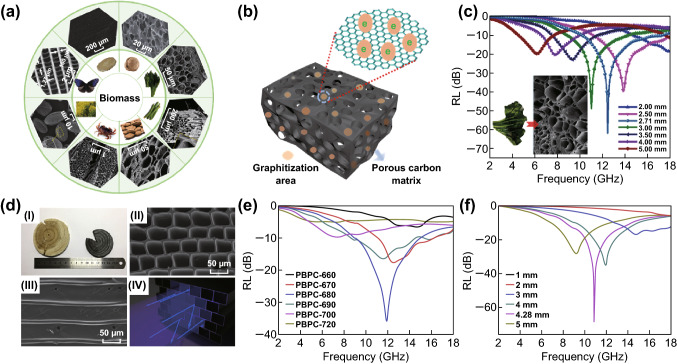
**a** Various biomass-derived PC materials prepared by a direct pyrolysis process. Reprinted with permission from Refs. [34, 51–56]. **b** The schematic illustration of graphitized PC. **c** The EM wave absorption properties of spinach stem-derived PC. Reprinted with permission from Ref. [34]. **d** (I) The digital camera photograph of wood and PBPC, (II, III) SEM images of radial and axial section for PBPC, (IV) The schematic representation of microwave absorption mechanism of PBPC. **e** The calculated reflection loss of PCBC samples with different annealing temperature at 4 mm. **f** The calculated reflection loss of PBPC-680 at different thicknesses. Reprinted with permission from Ref. [51]

Direct carbonization is the most facile and widely adopted approach for producing PC from biomass. Typically, the biomass precursor is subjected to pyrolysis under inert gas atmosphere at elevated temperature. After removal of volatile constituents (CH_4_, CO_2_, and some organics), the carbon product could be collected [49]. Meanwhile, the intriguing porous architectures in biomass will be retained in the final product after pyrolysis. As shown in Fig. 2a, PCs have been produced from a wide range of biomass resources via one step pyrolysis method, such as walnut shell [50], spinach stem [34], wood [51], rapeseed flower [52], bamboo [53], peanut shell [54], apricot shells [55], and morpho-butterfly wing scales [56]. Due to the diversity of biomass, the resultant pore morphology and size of PC rely strongly on the texture of gathered biomass. These developed pore structures could decrease effective dielectric values and promote the impedance matching. The optimal impedance would make the incident EM wave enter the interior of media for subsequent attenuation. In thermal treatment process, the high-temperature calcination accelerates the conversion of *sp*^3^ C-X (X: C, O, H, etc.) bond into aromatic *sp*^2^ C=C bond and hence resulting in the generation of graphitized carbon (Fig. 2b) [57, 58]. Along the graphite plane, the C=C bond links with each other forming a two-dimensional plane. The movement of numerous free electrons along the plane significantly boosts the electronic conductivity of biomass-derived PC. According to free electron theory: *ε*″ = 1/2*πρfε*_0_, where *ρ* is the resistivity and *ε*_0_ is the permittivity of vacuum [59–61], it can be deduced that the increase in conductivity would enhance the dielectric loss and microwave loss capacity of material. Besides, small amounts of heteroatoms (N, O, P, etc.) may be preserved in the carbon matrix after carbonization of biomass. On account of the different electronegativity between carbon atom and heteroatom, these heteroatoms could act as the polarization center by alternating EM fields, inducing dipole polarization and electronic polarization [62].

The enhanced polarization loss would attenuate the incoming EM energy, boosting the microwave absorption properties of materials. As a result, the biomass-derived PC possesses excellent dielectric property and microwave absorption capacity.

Wu et al. [34] prepared a hierarchical PC product with two-level porous structure from biomass spinach stem (Fig. 2c). The microsized cavities will work as dihedral angles to cause the reflection of EM wave within the material. This would extend the transmission path of EM wave and provide more opportunities to attenuate incident EM wave. The existence of nanosized pores in PC can reduce the effective permittivity and improve the impedance matching. The special hierarchical design and developed pore structure endow the PC medium with remarkable microwave absorption. An intensive reflection loss (RL) of − 62.2 dB can be obtained at thickness of 2.71 mm. In porous biomass-pyrolyzed carbon (PBPC) based on natural wood, the PBPC inherits the original shape of the wood and displays the regularly aligned parallel channel structure (Fig. 2d, I-III), which is rarely found in artificial materials [51]. Thanks to its highly oriented arrangement pore texture, there is minimal reflection of incident microwave on the side walls of channel, while most microwave will enter in via the channels (Fig. 2d IV). By pyrolyzing under various temperatures, the PCBC samples exhibit different attenuation capacity with EM energies (Fig. 2e). It is clear that the RL values of PCBC specimen prepared at temperature of 680 °C (PCBC-680) are much higher than those of other samples. The maximum RL value is up to − 68.3 dB with broad frequency bandwidth of 6.13 GHz at thickness of 4.28 mm (Fig. 2f). When the annealing temperature is higher or lower than 680 °C, the obtained sample shows inferior microwave absorption properties. Therefore, thermal treatment temperature is a crucial condition for determining the dielectric properties of sample. The higher temperature results in good conductivity and exorbitantly high dielectric value, which is unfavorable for impedance matching. On the contrary, low temperature would lead to weak attenuation capacity. Hence, an appropriate annealing condition is important factor for the eventual microwave absorption performance of PC sample.

### Activation Method

For PC materials, increasing surface area and porosity is required approach to optimize EM wave absorption performance. Tailoring of pore structure in biomass-derived PC can be fulfilled by chemical methods. Typically, activation method is a well-established and efficient route to punch pores into carbon media [63–65]. The PC prepared by activation method would usually have 4–50 times higher surface area than these non-activated PC [66].

Conventional activation methods include physical activation and chemical activation. With regards to physical activation, the biomass would be first carbonized into carbon component at relatively low temperature (usually < 800 °C). Then, the resultant carbon undergoes an activation process at higher temperature in the presence of suitable activator, such as CO_2_, air, and steam [67–69]. Owing to the small molecular size of these activators, the generated pore architectures by physical activation are at micropore level with the narrow distribution of pore dimension [70]. Zu’s group [71] highlighted that the number and size of micropores would increase with the activation time of CO_2_ extending. Similarly, Liu’s work [72] further reveals that the specific surface area and pore distribution could be easily controlled by regulating the CO_2_ activation duration.

For chemical activation, the whole reaction could be processed in one single procedure. Specifically, the carbon precursor is uniformly blended with activated agent through impregnation or grind method, followed by annealing at proper temperature under inner gas atmosphere [73]. Common chemical activators employed in reaction process includes ZnCl_2_ [74, 75], H_3_PO_4_ [76], and KOH [77, 78], etc. Among them, KOH activation is a well-developed method for introducing pores into biomass-derived carbon materials because of its mild activation temperature, higher production, and developed porosity with larger surface area (up to 3000 m^2^ g^−1^) [79]. Since the KOH activation method was first developed in 1978, it has been extensively applied in many experiments. Even till now, the mechanism for KOH activation is still unclear. Generally, the involved plausible reactions between KOH and carbon at elevated temperature are listed as Eqs. 3–7 [53]:

3 $$6{\text{KOH}} + 2{\text{C}} \to 2{\text{K}} + 3{\text{H}}_{2} + 2{\text{K}}_{2} {\text{CO}}_{3}$$

4 $${\text{K}}_{2} {\text{CO}}_{3} \to {\text{K}}_{2} {\text{O}} + {\text{CO}}_{2}$$

5 $${\text{CO}}_{2} + {\text{C}} \to 2{\text{CO}}$$

6 $${\text{K}}_{2} {\text{CO}}_{3} + 2{\text{C}} \to 2{\text{K}} + 2{\text{CO}}$$

7 $${\text{K}}_{2} {\text{O}} + {\text{C}} \to 2{\text{K}} + {\text{CO}}$$

The reaction first begins with solid–solid reaction at 400–600 °C, i.e., the KOH reacts with C, generating K_2_CO_3_ compound (Eq. 3). At ca. 600 °C, the KOH is exhausted completely. With temperature exceeding 700 °C, the K_2_CO_3_ is decomposed into potassium oxide (K_2_O) and carbon dioxide (CO_2_) (Eq. 4) and is absolutely consumed above 800 °C. Additionally, the produced K compounds and CO_2_ could further react with carbon over 700 °C. Namely, CO_2_ is reduced into CO by C component (Eq. 5), while K_2_CO_3_ and K_2_O are also reduced by C, forming metallic potassium (Eqs. 6, 7). The resultant PC sample usually contains some inevitable impurities such as metallic potassium and its compound, which should be removed by water and diluted HCl solution. During this process, the alkaline substance erodes the carbon surface and interior at high temperature. The induced irreversible expansion of carbon lattices results in the generation of developed porosity [65]. Based on preceding theory, the large specific surface area and increased porosity would promote the development of biomass-derived PC toward lightweight and high-efficient EM absorber.

**Fig. 3:**
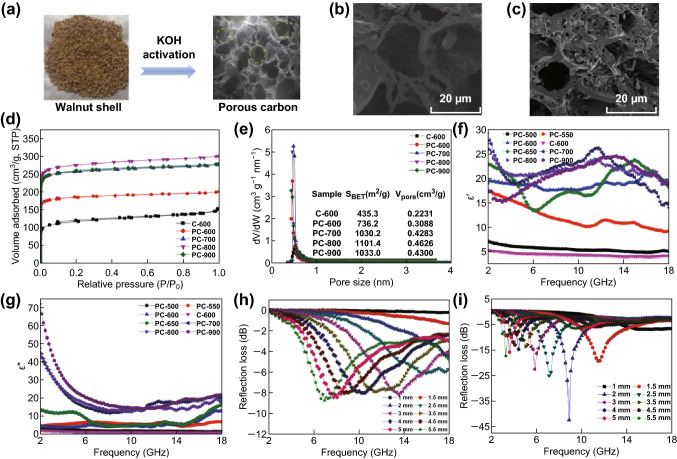
**a** Schematic illustration of the fabrication of walnut shell-derived porous carbon. SEM image of **b** C-600 and **c** PC-600. **d** Nitrogen adsorption–desorption isotherms, **e** pore size distributions, **f** real part of complex permittivity, and **g** imaginary part of complex permittivity of PC samples prepared under different conditions. The inset of **e** corresponds to the BET surface area values (*S*_BET_) and pore volume value (*V*_pore_) of all samples. **h** The RL value of C-600. **i** The RL value PC-600. Reprinted with permission from Ref. [80]

Qiu et al. [80] adopted the KOH activation technology to boost the microwave absorption capacity of walnut shell-derived PC, as shown in Fig. 3a. In Fig. 3b, c, it is obviously seen that the PC prepared by KOH activation (PC-600, *S*_BET_ = 746.2 m^2^ g^−1^) possesses more developed pore structure than non-activated PC (C-600, *S*_BET_ = 435 m^2^ g^−1^). By regulating the activation temperature, tunable surface area and pore volume could be easily realized, which significantly affect the complex permittivity of samples (Fig. 3d–g). It is noteworthy that PC with high surface area has large *ε*_*r*_ values, which is in contrast to the conclusion achieved in MG theory. Such abnormal phenomenon could be well explained by the enhanced polarization abilities and electrical conductivity of the materials. Benefiting from the increased dielectric properties and favorable porous structure, the strong microwave absorption intensity of − 42.4 dB at 8.88 GHz can be achieved at a thickness of 2 mm (Fig. 3i). The performance is much better than that of the non-activated samples (Fig. 3h). Similarly, our group prepared the PC sample from wheat flour through KOH activation method [81]. By controlling the period of activation, a nanoporous carbon with a three-dimension network architecture and a specific surface area of 1486.8 m^2^ g^−1^ can be obtained. Three-dimension (3D) network could generate induced currents along the skeleton under alternating EM fields. The presence of pore structure on skeleton forms the capacitor-like structure. Such long range-induced currents quickly decay in the resistive 3D network and are transformed into Joule heating, which lead to rapid consumption of massive incoming microwave. With the ultralow filler content of 8 wt%, the EM wave absorbency of − 51 dB was achieved at 1.8 mm. Meanwhile, the effective frequency bandwidth is up to 6 GHz at filler loading of 9 wt%. These extraordinary performances prove that the sample can be lightweight, highly efficient, and sustainable absorber.

### Template Method

The above two segments showed that large surface area and high porosity result in improved EM wave absorption properties. However, it is difficult to demonstrate the effect of pore size and distribution on microwave absorption behavior. The relevant investigation in depth would benefit the desired porous texture of goal absorber. Prior to the investigation of the role of pore size and distribution on attenuating EM energies, it is essential to prepare the PC sample with controllable pore structure. The above described activation technique is unable to accurately control the porosity of the structure, which is unfavorable for studying of relationship between pore architecture and microwave absorbency.

The template method is a powerful tool to synthesize biomass-derived PC with tailored pore dimension and distribution. The involved hard and soft templates are both vital to the synthesis of PC materials with regulable pore dimension and surface area [82–85]. As for template method, the technology is grounded on the self-assemble of block copolymers, surfactants, organic compounds, and so forth [86, 87]. The surfactant Pluronic^®^ F127 is a typical soft template. White et al. [88] reported the ordered porous carbon synthetized from fructose with the help of F127. The hydrophobic cores of the micelles, formed by polyphenylene oxide chains of F127, are the source of pores. However, the micelle is unstable at high temperature. At low temperature, most of biomass is hard to interact with surfactant, leading to low efficiency. Moreover, the high cost of soft template strictly restrains its wide application [84].

Compared with soft template technology, the hard template is more efficient and economic [89]. In the process, the artificial porous solid would be infiltrated with biomass solution. Subsequent, the biomass-template mixture undergoes the process of dehydrates and polymerizes/carbonizes. The final PC sample could be obtained by following removal of the initial template. Various inorganic materials can be adopted as hard templates, including SBA-15, alumina membrane, silica spheres, etc. [90, 91]. There have been reported studies on conversion of biomass into PC with controllable pore structure through hard template approach [84, 92]. Recently, Yin’s work adopted an in situ Stöber approach for design of hollow carbon microsphere with mesopore (HCMS) and hollow carbon microspheres (HCM) without mesopores [26]. The microwave absorption properties are significantly different due to their presence of mesopores. RL of − 84 dB was recorded for HCMS, and it is almost four times higher than that of the HCM. The remarkable example opens a significant avenue for the controllable synthesis of pore structure to optimize EM wave absorption property. It is a pity that the carbon source in the work is an organic reagent that includes environmentally harmful formaldehyde and resorcinol. Nonetheless, few studies have reported on synthesis of PC from nature biomass through hard template for EM absorption application, which may result from the low solvability of biomass in conventional reagent. Even so, biomass is the important renewable carbon-containing source in the world, and thus, the development of biomass-based PC will make great contributions to the progress of efficient and economic microwave absorber. Therefore, continuous research in depth about the preparation of PC with tunable pore size and distribution from biomass in the future is of great significance.

## Biomass-Derived PC-Based Composite Absorber

It is well known that the RL value is a crucial criterion used to assess the microwave absorption performance of a material. For satisfying practical application, the RL is required below − 10 dB, implying that more than the 90% of incoming EM wave could be absorbed and attenuated. Based on the transmission line theory, the RL can be expressed by Eqs. 8 and 9 [93, 94]:

8 $$Z_{\text{in}} = Z_{0} \sqrt {\mu_{r} /\varepsilon_{r} } \tanh [j(2\pi fd/c)]\sqrt {\mu_{r} /\varepsilon_{r} }$$

9 $${\text{RL}}\,({\text{dB}}) = 20\log |(Z_{\text{in}} - Z_{0} )/(Z_{\text{in}} + Z_{0} )|$$

where *Z*_in_ relates to the input impedance; *ε*_r_ and *μ*_r_ represent the complex permittivity and permeability; *f* is the EM wave frequency; *d* is the thickness of absorber; *c* is the velocity of light; *Z*_0_ is the impedance of free space. When the thickness is limited by the total weight, the RL highly depends on the EM parameters (i.e., *ε*_*r*_ and *μ*_*r*_) in the measured frequency range of 2–18 GHz. Hence, tailoring of *ε*_*r*_ and *μ*_*r*_ is an important prerequisite for superior EM absorbing properties. According to loss mechanism, the functional microwave absorbers could be divided into two categories: dielectric materials and magnetic materials [95]. Constructing multicomponent composites provides a way for designing the desired *ε*_*r*_ and *μ*_*r*_, enhancing the multiple loss, which achieves the remarkable microwave absorption [96–99].

In general, the dielectric loss ability primarily originates from polarization and conductivity loss [100]. The polarization loss could be further divided into interfacial polarization, electronic polarization, dipolar polarization, and ionic polarization. Electronic and ionic polarization usually appear at a much higher frequency range of 103–106 GHz, which can be excluded in microwave range [101]. Therefore, interfacial and dipolar polarization should be the main relaxation attenuation mechanism in 2–18 GHz. Generally, the interfacial relaxation process always occurs in a heterogeneous system. The accumulation and uneven distribution of space charges at the interfaces will produce a macroscopic electric moment that can decay the incident EM energy effectively. The dipolar polarization occurs in molecule with obvious dipole moment. According to Debye polarization equation, these relaxation processes could be estimated by analyzing the relationship between *ε*″ and *ε*′, as shown in Eq. 10:

10 $$\left( {\varepsilon^{{\prime }} - \frac{{\varepsilon_{s} + \varepsilon_{\infty } }}{2}} \right)^{2} + (\varepsilon^{{\prime \prime }} )^{2} = \left( {\frac{{\varepsilon_{s} - \varepsilon_{\infty } }}{2}} \right)^{2}$$

where *ε*_*s*_ is the static permittivity and *ε*_∞_ is the relative permittivity at high-frequency limit. From Eq. 10, it is deduced that the plots of *ε*′–*ε*″ should be a semicircle (generally regarded as Cole–Cole semicircle). Each semicircle represents one Debye relaxation process [45]. More Cole–Cole semicircles indicate the strong polarization relaxation process during attenuating EM wave process. The magnetic loss is definitely another key factor for the microwave absorption. It is widely known that the magnetic loss is contributed dominantly by eddy current loss, exchange resonance, and natural resonance in the microwave frequency band [102]. The resonant peaks at low-frequency and high-frequency regions are usually associated with the natural resonance and exchange resonance, respectively. The contribution of eddy current to magnetic loss can be assessed by analyzing the dependence of *μ*″ (*μ*′)^−2^ f^−1^ on frequency. If the *μ*″ (*μ*′)^−2^ f^−1^ value keeps constant with the variation of frequency, the eddy current loss will be the main reason for the magnetic loss [103]. To evaluate the contribution of dielectric and magnetic loss in attenuating EM energies, the calculated dielectric loss tangent (tan*δ*_*ε*_ = *ε*″/*ε*′) and magnetic loss tangent (tan*δ*_*μ*_ = *μ*″/*μ*′) of absorber can be used to assess the loss ability of the materials in dissipating EM wave energy [94]. It is well known that the absorbers with higher tan*δ*_*ε*_ and tan*δ*_*μ*_ values usually have better EM wave absorption, which assures the incoming EM wave to be consumed quickly through the absorber materials.

As for biomass-derived PC, the loss mechanism is mainly resultant from limited dielectric loss. The EM wave absorption properties are insufficient to broaden their applications. Hence, the incorporation of other functional materials into biomass-derived PC is an effective strategy to boost its microwave absorbency.

### Biomass-Derived PC-Based Binary Composite Absorber

Guan and his co-worker [104] decorated jackfruit peel-derived PC with Ni(OH)_2_ nanosheet for high-performance microwave absorption application. The Ni(OH)_2_/PC composite exhibits the good microwave absorption properties with RL value of − 23.6 dB at 15.48 GHz. The increased dielectric properties are attributed to enhanced interfacial polarization and porous structure. Compared with dielectric materials Ni(OH)_2_, the magnetic substance such as magnetic metals (e.g., Fe, Co, Ni, and their related alloys) and/or metal oxides (e.g., γ-Fe_2_O_3_, Fe_3_O_4_, and CoFe_2_O_4_) may be better alternatives [105–108]. Incorporating magnetic materials into biomass-derived PC not only enhances interfacial polarization, but also gains the favorable magnetic loss. To this end, numerous biomass-derived PC-based magnetic composites with superior microwave absorption performance have been reported in recent years.

**Fig. 4:**
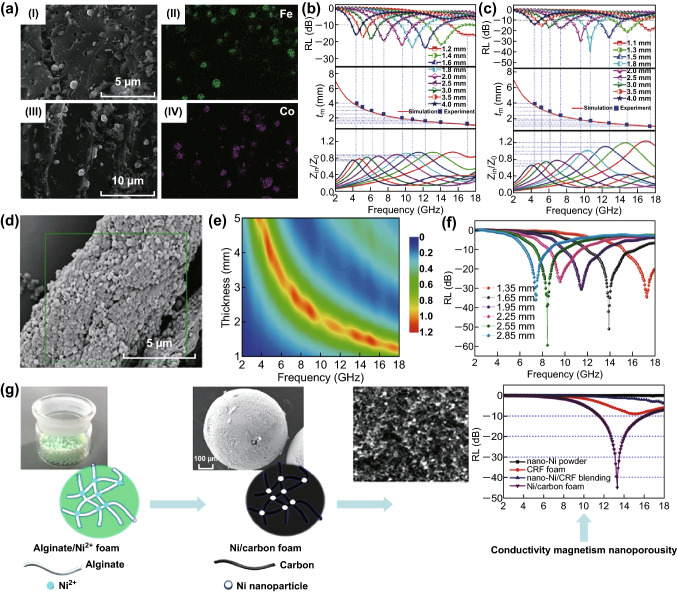
**a** SEM image and corresponding elemental mapping information of (I, II) RHPC/Fe and (III, IV) RHPC/Co; microwave absorption performance of **b** RHPC/Fe and **c** RHPC/Co. Reprinted with permission from Ref. [109]. **d** SEM image of Co/C composite. **e** Impedance matching contour of Co/C composite. **f** The RL value of Co/C composite. Reprinted with permission from Ref. [111]. **g** Procedure for the preparation of the Ni/carbon foam and its EM wave absorption performance. Reprinted with permission from Ref. [112]

Using rice husk-derived porous carbon (RHPC) as substrate, Fang et al. [109] imbedded the Fe and Co magnetic nanoparticles into RHPC matrix for EM wave attenuation application (Fig. 4a). The obtained RHPC/Fe and RHPC/Co both exhibit the high tangent dielectric and magnetic loss values. The synergistic effect between dielectric loss and magnetic loss endows the composites with strong EM wave dissipation ability. At thin thickness of 1.4 mm, the RHPC/Fe exhibits a RL value of − 21.8 dB with broad frequency bandwidth of 5.6 GHz (Fig. 4b), and the strong microwave absorption intensity − 40.1 dB was obtained at 1.8 mm for RHPC/Co composite (Fig. 4c). Li et al. [110] reported a flexible two-step method consisting of immersion and subsequent carbothermal reduction under N_2_ atmosphere for fabrication of Co/C fibers as synergistic EM absorber by using nature cotton as raw materials. Appropriate amount of Co nanoparticles in carbon fiber generates a better dielectric and magnetic property as well as optimized impedance matching. As the result of these features, the Co/C fiber shows the remarkable microwave absorbing ability. At the filler content of 33%, the RL below − 10 dB could cover the frequency range of 11.3–18 GHz, which is almost the entire Ku-band (from 12 to 18 GHz). Similarly, our group also synthesized the Co/C composite using the cotton as porous carbon precursor and ZIF-67 as the magnetic metal Co source (Fig. 4d) [111]. As shown in Fig. 4e, the optimal impedance matching was achieved. This should be attributed to the synergy of relaxation, magnetic resonance, conductive loss. At the low filler loading of 25%, the maximum reflection loss can achieve − 60.0 dB at 8.48 GHz. Through adjusting the thickness to only 1.65 mm, the strong RL intensity is up to − 51.2 dB at 13.92 GHz with a broad bandwidth of 4.4 GHz (Fig. 4f). For Ni/C composite, Zhao et al. [112] reported an EM-functionalized Ni/C foam produced via an alginate/Ni^2+^ hydrogel (Fig. 4g). The fabricated foam has high porosities with large surface area of 451 m^2^ g^−1^, a moderate conductivity (6 S m^−1^), and important magnetism. Compared with traditional carbon foam and nano-Ni powder, the Ni/C foam with unique microstructure and special synergistic effects of multiple components maintains great EM wave absorption performance. When the filler content is only 10 wt%, the maximum RL value of − 45 dB was obtained at a thickness of 2 mm with effective frequency bandwidth of 4.5 GHz. Similarly, our group had incorporated the magnetic Ni nanoparticle into rice-derived PC matrix via a facile dipping method and subsequent activation process [113]. By controlling the precursor ratio, optimized microstructure and component could be simultaneously realized. The effect of developed pore structure and heterojunction generates the enhanced interface polarization. The 3D framework offers the transmission route of induced current. As a consequence of these properties, the as-prepared Ni/C composite shows significant enhancement in microwave absorption. At the low filler content of 15%, the enhanced RL of –52 dB and wide effective absorption frequency bandwidth of 5 GHz were realized.

In comparison with the introduction of magnetic metals, researchers are more apt to hybridize the ferrite with biomass-derived PC, owing to its low toxicity, high compatibility, and strong spin polarization at room temperature [32, 114, 115]. Gao et al. [116] decorate the Fe_3_O_4_ nanoparticles with walnut shell-derived porous carbon (WPC) with honeycomb-like through a facial solvothermal method. The obtained Fe_3_O_4_/WPC composite displays the better microwave absorbency as compared to pure Fe_3_O_4_ and single WPC. Its high-efficiency EM attenuation resulted from dielectric loss of lightweight conductive WPC and magnetic loss of Fe_3_O_4_ nanoparticles.

### Biomass-Derived PC-Based Ternary Composite Absorber

**Fig. 5:**
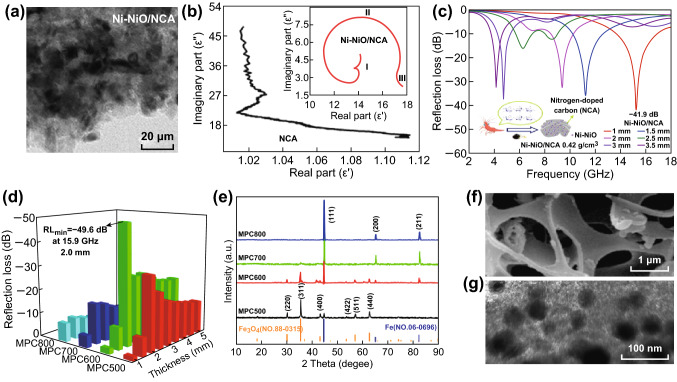
**a** TEM image of Ni-NiO/NCA composite. **b** The Cole–Cole semicircle of NCA and Ni-NiO/NCA composite. **c** The microwave absorption performance of Ni-NiO/NCA composite. Reprinted with permission from Ref. [117]. **d** Column chart of RL values for MPC composites with various carbonized temperatures. **e** XRD profiles of all MPC composites. **f** SEM image and **g** TEM image of MPC-600. Reprinted with permission from Ref. [118]

Beyond such two-phase composites, ternary composites based on biomass-derived PC have also attracted immense interests. Their attracted features, like multiple interfacial polarization and superior impedance matching, would further boost the EM attenuating capacity of biomass-derived PC. Wang et al. [117] embedded the Ni–NiO nanoparticles into chitosan-derived nitrogen-doped carbon aerogel (NCA) via an explosion method (Fig. 5a). From Fig. 5b, it is observed that the as-prepared ternary composites Ni-NiO/NCA possess much more Cole–Cole semicircles than pure NCA substance, indicating the enhanced Debye polarization process induced by multiple heterostructure. Strong RL intensity of − 49.1 dB was obtained at thin thickness of 1 mm (Fig. 5c). Wang et al. [118] investigated the EM response behavior of diverse magnetic hierarchical porous carbon (MPC) composites prepared at different annealing temperature using loofah sponge biomass and Fe(NO_3_)_3_·9H_2_O as ingredients. From Fig. 5d, it is obvious that the sample obtained at 600 °C (MPC-600) possesses much better microwave absorption properties compared to other samples. The author ascribes the great difference in EM absorption performance to the variable component of samples. In detail, the MPC-500, MPC-700, and MPC-800 samples are binary composites according to XRD results (Fig. 5e). The former is comprised of Fe_3_O_4_ and carbon, and the latter two is composed of Fe and carbon component. The MPC-600 is a typical ternary component, including Fe and Fe_3_O_4_ as well as carbon media. Integrating the multiple interfaces, unique porous structure, and magnetic loss (Fig. 5f, g), the MPC-600 displays superior EM loss property as compared to other samples. A RL value of − 49.6 dB is obtained at thin thickness of 2 mm, and effective frequency bandwidth is up to 5 GHz. Zhao et al. [119] demonstrated a ternary composite of Co@crystalline carbon@carbon aerogel that is produced from biomass alginate. An enhanced microwave absorption of − 43 dB was achieved under filler loading of 10 wt%. In this work, the author deduced that the multiple interfacial polarization at interface of neighboring phase with different dielectric constants, such as, Co nanoparticles/crystalline carbon shells, amorphous carbons/crystalline carbon layers, and carbon framework/wax, should be responsible for the enhanced dielectric properties.

## Conclusion and Perspectives

Microwave absorption materials are desired to come with strong absorption intensity, broad absorption frequency bandwidth, lightweight, thin thickness, as well as low cost, high production, and ease of processing. In this review, biomass-derived PCs have demonstrated huge potential to meet such requirements, due to its low density, facile synthesis strategies, tunable EM properties, as well as abundant available precursor. In this review, we systematically summarized the key factors determining the EM loss capacity of biomass-derived PC media, and highlighted promising approaches have shown to improve the EM absorption properties of biomass-derived PC materials. Structural modification and compositional design have been demonstrated to be instrumental in achieving excellent EM absorption properties. For PC synthesized directly via carbonization of biomass, thermal treatment condition is a significant factor for the enhancing microwave attenuation property. The activation method and templating method are both feasible strategy in tailoring the pore structure of biomass-derived PC to achieve remarkable EM properties. Furthermore, their performance could be further improved by hybridizing with other functional materials through synergizing multiple loss mechanisms.

Table 1 summaries the microwave absorption properties of current reported biomass-derived PC-based absorber. These materials indeed have made remarkable achievement. Even so, there are some challenges that still hinder its development. On one hand, it is certain that enlarging surface area and adjusting pore structure are favorable for enhancing the microwave absorption performance of biomass-derived PC materials. However, effect of pore size and pore distribution on microwave absorption behavior is still unclear, which hinders the design of PC with better performance. The issue remains to be further investigated. Along with this issue, another challenge is to develop an effective approach to fabricate PC materials with controllable pore structure from biomass resource. It is acceptable that the construction of multicomponent composite is a promising strategy to broaden the application of biomass-derived PC in microwave absorption and has made a great achievement. Nevertheless, there are still some fundamental issues to be addressed, i.e., understanding the absorption mechanisms in depth for the multicomponent systems and investigating the effect of interface species on the absorption performance. Considering a practical application, apart from the characteristics such as lightweight, low cost, and remarkable absorption, the features of anti-causticity, good thermal stability, and hydrophobicity should be also considered due to the possible harsh working environment of electric devices. Hence, exploring versatile biomass-derived absorbing materials with extensive requirements such as well resistant to high temperatures, good hydrophobicity, and strong anti-causticity should be put forward. Finally, apart from the porous structure, biomass in nature offers numerous advantages in designing nanocomposites. The combination of bio-inspiration, nanotechnology, and chemical synthesis is expected to generate an increase in nanostructured materials from renewable sources for microwave absorption application in the years to come.

**Table 1:** Recent progress in biomass-derived PC-based EM wave absorption material

Sample	Minimum RL value		Filler content (%)	RL ≦ − 10 dB		References
	*d*_m_	RL_min_		*d*_m_	*f*_e_	
Spinach stem-derived PC	2.71	− 62.2	30	2.71	7.3	[34]
PBPC	4.28	− 68.3	–	3.73	7.63	[51]
PC	2	− 42.8	70	1.5	2.24	[80]
Nanoporous carbon	2.9	− 51	8	1.8	4.8	[81]
AC/Ni(OH)_2_	6	− 23.6	50	4.5	2	[104]
RHPC/Fe	1.4	− 21.8	25	1.4	5.6	[109]
RHPC/Co	1.8	− 40.1	25	1.8	2.7	[109]
Co/C fiber	2	− 31	33	2.5	6.7	[110]
Carbon-cotton/Co@NPC	2.55	− 60.0	25	1.65	4.4	[111]
Ni/C foam	2	− 45	10	2	4.6	[112]
HPMC	1.7	− 52	15	1.65	5	[113]
Fe_3_O_4_/WPC-600	–	− 51.3	50	2	5.8	[116]
Co@crystalline carbon@carbon	1.5	− 43	10	1.7	4.6	[119]
Ni-NiO/NCA	1.5	− 41.9	50	1.5	3.5	[117]
Amorphous PC/Fe_3_O_4_@Fe	2	− 49.6	30	2	5	[118]
